# Evaluating Interleukin-2 and Its Receptors As Indicators of Acute Renal Graft Rejection

**DOI:** 10.7759/cureus.73185

**Published:** 2024-11-07

**Authors:** Athina Gompou, Despoina N Perrea, Theodore Karatzas, Anastasia Kastania, Aikaterini Dimaki, Emmanouil M Xydias, Ioannis Boletis, Alkiviadis Kostakis

**Affiliations:** 1 Department of Nephrology and Renal Dialysis, IASO Thessaly, Larissa, GRC; 2 Department of Nephrology, Transplantation Unit, Laiko General Hospital of Athens, Athens, GRC; 3 Department of Experimental Surgery and Surgical Research, National and Kapodistrian University of Athens School of Medicine, Athens, GRC; 4 Department of Propaedeutic Surgery, Laiko General Hospital of Athens, Athens, GRC; 5 Department of Informatics, School of Information Sciences and Technology, Athens University of Economics and Business, Athens, GRC; 6 Department of Obstetrics and Gynecology, Aristotle University of Thessaloniki, Thessaloniki, GRC; 7 Department of Biostatistics, Biomedical Research Foundation Academy of Athens, Athens, GRC

**Keywords:** acute graft rejection, cystatin-c, interleukin 2, prognostic biomarker, renal transplant surgery, soluble interleukin-2 receptor

## Abstract

Introduction

Interleukin-2 (IL-2) is a cytokine that exerts its actions via binding to a variety of interleukin-2 receptors (IL-2R), thereby stimulating T-cell response. Acute renal graft rejection (AR) is known to be mediated by CD8+ T-cells, through the IL-2 pathway. The aim of this study was to determine whether IL-2 and IL-2R could work as prognostic biomarkers of AR.

Methods

IL-2, IL-2R and Cystatin-C levels were measured in the serum of 50 patients who underwent a kidney transplant, once pre-operatively and at four different time points post-operatively (second, sixth, 14th day and third month). Of the total number of patients, ultimately 10 (20%) had an episode of AR.

Results

No statistically significant difference in IL-2 levels was found between those who experienced AR and those who did not, at any of the studied time points. On the other hand, measurement of IL-2R levels on the sixth and 14th day post-operatively showed that people with AR had a statistically significant increase in its value compared to patients who did not have an AR episode (p=0.027 and p=0.019, respectively). In addition, comparing the values ​​of IL-2R with that of Cystatin-C in different time periods, it was found that there is a significant positive linear correlation on the second and sixth postoperative day between the values ​​of the associated parameters (r=0.280, p=0.049 and r=0.372, p=0.008 respectively).

Conclusion

The measurement of IL-2R from the sixth to 14th postoperative day could be used as a reliable prognostic biomarker of AR, however additional studies and standardised diagnostic thresholds are required before the routine clinical application is feasible.

## Introduction

Renal transplantation is a challenging procedure, which is associated not only with surgical, but also graft-related complications, known to adversely affect graft health and ultimate procedure success, with the most prominent being acute graft rejection (AR). In order to accurately identify and effectively address such complications in a timely manner, close, attentive patient monitoring is mandatory and in particular a reliable biomarker that can be consistently measured in the patient’s serum. Such markers currently used in clinical practice are serum creatinine (sCr) and Cystatin-C, however, there are some limitations regarding their reliability [1,2]. Namely, sCr levels, being the traditional means of estimating kidney function, are known to be affected by the patient’s age, gender, muscle mass and liver function, as well as by several medications and diseases [3]. Cystatin-C has been proposed as an alternative prognostic marker to the traditional sCr, demonstrating a statistically significant, very strong correlation to sCr levels (r=0.969, P<0.01) post-transplantation, as we demonstrated in a previous study [4]. In the same study, the estimated glomerular filtration rates (eGFR) calculated with either sCr or Cystatin-C were again significantly correlated (r=0.896, P<0.01), indicating the applicability of Cystatin-C as a marker of renal function [4]. However, the use of Cystatin-C as a biomarker does have certain limitations, as its levels may be deceptively elevated in older men, patients with increased muscle mass, hyperthyroidism and with the administration of corticosteroids [5,6]. Therefore, fluctuations in the levels of either sCr or Cystatin-C cannot always be reliably attributed solely to graft-related complications, thus affecting their utility and applicability as predictors.

Interleukin-2 (IL-2) is a protein produced by CD4+ T-cells following their stimulation by an antigen. It enhances the cytolytic activity of CD8+ T-cells and natural killer cells, an ability observed both in vitro and in vivo [7-9], via regulating the expression of proteins involved in their lytic activity [10]. Additionally, low doses of IL-2 are correlated with the activation of T-regulatory (Treg) cells, thus reducing autoimmune responses, an ability with considerable potential in the treatment of autoimmune diseases and Graft Versus Host Disease [11-13]. Upon CD4+ T-cell activation, IL-2 production rapidly increases, a process reflected in measured serum levels [14,15]. The receptor of interleukin-2 (IL-2R) is a protein expressed in a variety of immune cells, normally by Treg cells, but also upon activation of CD8+ T-cells. When an immune response is triggered, a part of the receptor, the soluble IL-2R (sIL-2R), is severed from the rest of the membrane-bound IL-2R and released in the serum [16,17]. The serum levels of sIL-2R (henceforth IL-2R) can therefore be used to monitor the activity of immune-related diseases [18], with promising applications in the prediction of renal outcomes in kidney-related immune conditions (IgA nephropathy) [19].

AR is known to be mediated by T-cells [20,21], triggering a local inflammatory process and in turn recruiting more T-cells and other inflammatory cells to move to the graft site, leading to an inflammation cascade and resulting in AR [22,23]. As already mentioned, activated T-cells can over-express IL-2R upon activation. Therefore, the determination of the serum levels of IL-2 and its receptor could prove to be a useful tool in the early recognition of a potential AR in kidney transplant patients. The aim of the present study is to examine the levels of these proteins at different time points in patients who have undergone kidney transplantation and in particular those with AR, in addition to examining their correlation to established biomarkers and their utility as AR predictors.

The present study is based on the findings of a doctoral dissertation that has been archived in the “Pergamos” Institutional Repository and Library of the National and Kapodistrian University of Athens, on September 21, 2015 (uoadl:1308022) [24].

## Materials and methods

Patients and transplantation protocol

All patients who underwent kidney transplantation at the Transplantation Unit of “Laiko” Hospital in Athens Greece, between the 27th of March, 2003 and the 16th of July, 2004 were enrolled in the study. No limitations were placed on the medical indications for transplantation and the type of donor utilized. Exclusion criteria were previous history of renal transplant and positivity for Hepatitis B (HBV), Hepatitis C (HCV) and cross-match testing. After the transplantation procedure, patients received triple immunosuppressive treatment consisting of cyclosporine A (CsA) or tacrolimus (FK), depending upon relative contraindication for CsA (e.g. hyperlipidaemia), mycophenolate mofetil (MMF), and methylprednisolone (MP). According to the protocol, the following doses were administered: (a) CsA 3 mg/kg/day with dose adjustments to obtain two-hour peak levels (C2 levels) of 700-800 ng/mL as measured in whole blood by radioimmunoassay (mRIA Instar by Sorin, Arvada, Colorado, USA); (b) FK 0.05 mg/kg/day with a target level of 7-8 ng/mL; and (c) MMF 2 g/day. A single shot of 500 mg of MP was given intraoperatively and 20 mg/day thereafter, with gradual dose tapering at a rate of 2 mg per week. This protocol was supplemented with additional medications, based on individual patient circumstances. Urine samples were collected to exclude possible urinary tract infections. Written, informed consent was obtained from all patients for participation in and publication of the study. The study was approved by the National and Kapodistrian University of Athens, School of Medicine Bioethics and Conduct Committee (approval no.: 12139) and the Scientific Council of the “Laiko” General Hospital in Athens, Greece, where the study took place (protocol number S.C. 420).

IL-2 and IL-2R measurements

Several investigations and measurements were performed for the purposes of the original doctoral dissertation [24], however for the purposes of the present study, only Cystatin-C, IL-2 and IL-2R measurements will be described. Blood samples were collected from each patient after hemodialysis prior to graft implantation (pre-Tx) and after transplantation (post-Tx) at two, six, 14 and 90 days. All samples were collected in plastic tubes (BD Vacutainer, Oxford, United Kingdom) and were left at room temperature for 30 minutes. They were then centrifuged at 3000 rpm for 15 minutes and the serum was frozen at -80°C. Enzyme-linked immunosorbent assay (ELISA) was performed for the measurement of all three markers, using a BIORAD Model 680 microplate reader (Bio-Rad Laboratories, Inc., Hercules, USA) for all photometric assessments. Serum Cystatin-C was measured using the Quantikine DSCTC0 ELISA kit (R&D Systems, Minnesota, USA). Cys-C values were obtained using a particle-enhanced immunoturbidimetric assay in a Hitachi 717 autoanalyzer (Hoffmann-La Roche, Basel, Switzerland) using reagents from Dako Diagnostics. Reference values for serum levels of Cystatin-C were 0.6-1.3 mg/L. Serum IL-2 and IL-2R were assessed using the corresponding ELISA kits (Catalog No45, ALPCO Diagnostics, New Hampshire, USA).

Statistical analysis

Data analysis was performed using the Statistical Package for the Social Sciences (IBM SPSS Statistics for Windows, IBM Corp., Version 19.0, Armonk, NY). All data are presented as mean ± SD. All numeric data was checked for normal distribution using the Kolmogorov-Smirnov test. The student's t-test (for normally distributed data) or the Mann-Whitney U (Wilcoxon) statistic (for non-normally distributed data) were used to examine differences between the groups of patients who experienced acute rejection and those who did not, in addition to differences in pre- and post-Tx marker levels. For correlation assessment between normally distributed variables, the Pearson correlation coefficient was used. Otherwise, the non-parametric Spearman Rho correlation coefficient was calculated. Results with P-values lower than 0.05 were considered statistically significant.

## Results

Ultimately, a total of 50 patients were enrolled in the present study. Of those, 18 were females (36%) and 32 males (64%), aged between 15 and 70 years (mean age = 42.5 years). Of the enrolled patients, 32 received transplants from a living donor (relative) and 18 from a deceased donor. The donor's mean age was 43.5 years. All donors were HBV- and HCV-, with the exception of one donor who tested positive for HCV. All patients had end-stage renal disease (ESRD) prior to transplantation and no procedure was pre-emptive. Patients’ average waiting time for transplantation was 8.5 years. The five most common causes of ESRD in the participants were in descending order: unknown aetiology, diabetic nephropathy, membranous nephropathy, focal segmental glomerulosclerosis and polycystic disease. No patient deaths occurred throughout the duration of the study. The renal transplant survival rate was 96% with two graft losses, one due to AR and the other due to delayed graft function. Kidney function was normal for 40 patients (80%), while 10 patients (20%) experienced AR. More specifically, eight patients (16%) experienced acute cellular rejection, one patient (2%) acute vascular-type rejection, and one (2%) was borderline suspicious for cellular rejection. The diagnosis of AR was based on standard clinical findings and was confirmed by percutaneous renal biopsy on the third post-Tx day. Finally, no patients had graft failure following treatment with anti-rejection therapy. The demographic characteristics and details regarding transplantation immunosuppression are summarized in Table 1.

**Table 1:** Patient demographic and transplantation-related characteristics. CSA: Cyclosporine A; MMF: mycophenolate mofetil; MP: methylprednisolone; Basiliximab (SIMULECT®); Daclizumab (ZENAPAX®); SIR: sirolimus; FK: tacrolimus; (0:1:2): number of patients with (zero:single:double) donor-recipient mismatch for each HLA antigen assessed; HLA: human leukocyte antigen

Parameter (unit)	Outcome
A - Demographic	
Patients (n)	50
Body weight (kg), mean±SD	69.3 ± 14.8
Age (y), mean±SD	42.5 ± 11.5
Gender (M:F)	32:18
Donor type (living: deceased)	32:18
Transplant type (primary:secondary)	50:0
Acute rejection (Yes:No)	10:40
B - HLA mismatches	
HLA-A (0:1:2)	1:41:8
HLA-B (0:1:2)	3:45:2
HLA-DR (0:1:2)	5:40:5
HLA-DQ (0:1:2)	15:24:11
HLA-CW (0:1:2)	20:28:2
C - Medications	
MMF, CSA, MD, SIMULECT	28
MMF, FK, MD, ZENAPAX	7
SIR, FK, MD, ZENAPAX	4
MMF, FK, MD, SIMULECT	3
FK, SIR, MD, ZENAPAX	3
MMF, SIR, MD, ZENAPAX	2
MMF, CSA, MD	1
MMF, CSA, SIMULECT	1
MMF, CSA, MD, SIMULECT/FK, SIR, MD, plasmapheresis, immunoglobulins	1

IL-2 demonstrated a significant increase compared to its levels pre-Tx, with the values remaining relatively stable on subsequent measurements. However, during all post-Tx follow-up measurements, levels maintained a statistically significant elevation compared to those pre-Tx. Post-Tx IL-2R levels followed an opposite trend, with a statistically significant decrease compared to those pre-Tx, which was maintained throughout the follow-up period. On the contrary, the post-Tx levels of IL-2R are statistically significantly lower than the pre-Tx value. The mean values ± standard deviation of IL-2 and IL-2R pre-Tx and post-Tx (second, sixth, 14th day and third month) for all patients are presented in Table 2.

**Table 2:** Mean ± standard deviation of IL-2 and IL-2R levels at different time points before and after kidney transplantation. Pre-Tx: pre-transplantation; Post-Tx: post-transplantation; IL-2: interleukin-2; IL-2R: interleukin-2 receptor; IL-2 reference range in healthy adults: 0.05 to 5.7 pg/mL; IL-2R reference range in healthy adults: 2132-7487 pg/mL; P-values are derived from the Mann-Witney U test and are with respect to the comparison with pre-Tx levels, *: statistically significant difference

Parameter (unit)	Pre-Tx	Post-Tx			
		2^nd^ day	6^th^ day	14^th^ day	3^rd^ month
IL-2 (pg/mL)	10.2 ± 2.2	16.5 ± 1.5 (P<0.001)*	15.9 ± 1.4 (P<0.001)*	17.4 ± 1.5 (P<0.001)*	15.4 ± 1.5 (P<0.001)*
IL-2R (pg/mL)	2248 ± 142	1233 ± 144 (P<0.001)*	1126 ± 148 (P<0.001)*	1262 ± 102 (P<0.001)*	954 ± 76 (P<0.001)*

No statistically significant difference in IL-2 levels was found between patients who experienced AR (AR+) and those who did not (AR-), at any time point of assessment. On the contrary, the levels of IL-2R demonstrated a statistically significant increase on the sixth and 14th post-Tx days in AR+ patients compared to AR- patients (p = 0.027 and p = 0.019, respectively). This difference is summarized in Table 3.

**Table 3:** Mean ± standard deviation of IL-2R values (pg/mL) at different time points, between AR+ and AR- groups. There appears to be a statistically significant difference between the two groups on the sixth and 14th postoperative days. Pre-Tx: pre-transplantation; Post-Tx: post-transplantation; IL-2R: interleukin-2 receptor; AR(+): patients with acute rejection; AR(-): patients without acute rejection; P-values are derived from the Mann-Witney U test and correspond to comparisons between the two groups at same time-points, *: statistically significant difference

Patient group	Pre-Tx	Post-Tx			
		2^nd^ day	6^th^ day	14^th^ day	3^rd^ month
AR+ patients (N=10)	2,367 ± 839	1,479 ± 876	2,350 ± 1,678	1,761 ± 665	1,029 ± 494
AR- patients (N=40)	2,219 ± 1055	1,172 ± 879	945 ± 574 (P=0.027)*	1,137 ± 684 (P=0.019)*	936 ± 558

**Figure 1:**
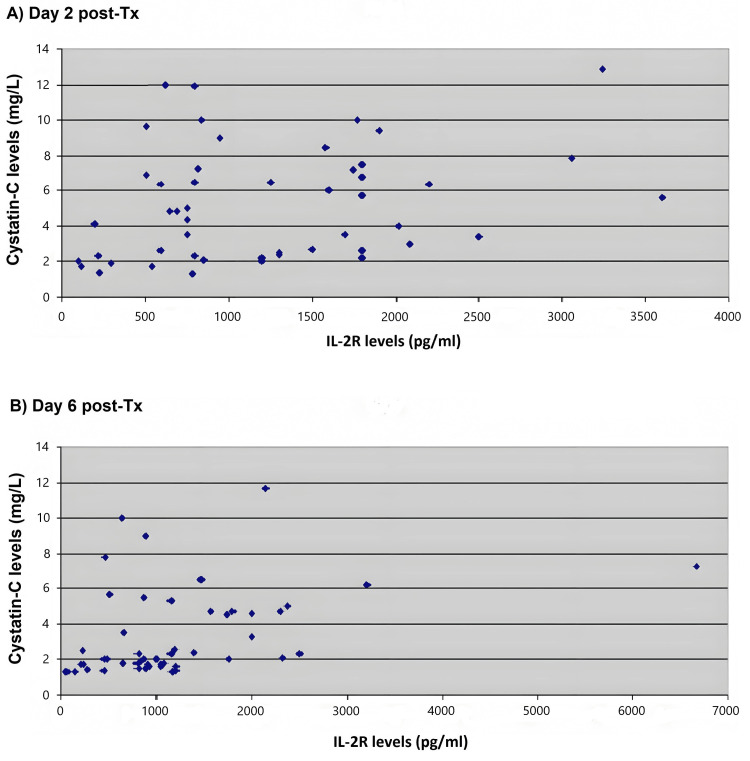
Correlation between IL-2R and Cystatin-C values on second and sixth post-Tx days. There is a statistically significant positive correlation between the values of the two parameters during (A) the second-day post-Tx (r=0.280, p=0.049) and (B) the sixth-day post-Tx (r=0.372, p=0.008). IL-2R: interleukin 2 receptor; post-Tx: post-transplantation

Furthermore, comparing the values of IL-2R with that of Cystatin-C at different time points, a statistically significant positive linear correlation between them was observed on the second and sixth post-Tx days (second day: r=0.280, p=0.049; sixth day: r=0.372, p=0.008). This observation is visually depicted in Figure 1. No statistically significant correlation was found between IL-2R and Cystatin-C levels at other time points.

## Discussion

The present study demonstrated the connection of serum IL-2 and IL-2R levels with graft and recipient interaction, in addition to demonstrating a potential role in predicting graft-related outcomes post-Tx. Namely, both biomarkers were significantly increased post-Tx in the serum of recipients, however, when the patients were stratified based on AR occurrence, IL-2R levels were significantly higher in the AR+ group on days 6 and 14 post-Tx. Simultaneously, IL-2R levels had a significant positive linear correlation to Cystatin-C levels during the same days. This is perhaps indicative of the potential of IL-2R levels as a prognostic factor for AR occurrence post-Tx.

AR represents an established risk factor for renal graft failure, occurring predominantly in the early post-Tx period, and it should be diagnosed quickly and treated appropriately. In current clinical practice, the diagnosis of AR is based on sCr or Cystatin-C levels. Unfortunately, this is not a perfect system, as these parameters have been shown to come with considerable limitations in certain patient subpopulations; whereby eGFR calculations based on them may be misleading or discordant [25-27]. Therefore, clinical monitoring of the renal transplantation patient based solely on them may be insufficient to reliably predict graft status and AR risk.

These parameters can be supplemented by the measurement of IL-2 and IL-2R levels in the patients’ serum. IL-2 and its receptor are proteins, whose levels indicate the activation of CD8+ T-cells [28]. IL-2 is known to increase the cytolytic activity of these cells through the expression of certain proteins [29]. IL-2R is expressed on the surface of activated T-cells and the levels of sIL-2R can be measured in the blood in order to assess T-cell and immune system activity [30,31]. It is also known that AR is mediated by CD8+ T-cells through a pathway that involves the inflammation cascade [32,33]. Therefore, it stands to reason that the determination of IL-2 and/or IL-2R in patients receiving a renal graft could be used as an indicator of AR.

IL-2 levels have been used for the prediction of AR before. Tefik et al. [34] reported higher IL-2 levels in patients with early rejection (on days 1 and 7) compared to successful transplantation (P = 0.042 and P = 0.031 respectively). IL-2 levels were also significantly higher in AR patients compared to successful cases in the study by Jin et al. [35]. These observations were consistent with the multicentre study by Millán et al. [36], who also demonstrated a statistically significant elevation of IL-2 levels in AR patients, amongst other potential risk prediction biomarkers. However, in the present study, IL-2 levels did not seem to follow the same trend, as there were no statistically significant differences between AR(+) and AR(-) patients, a discrepancy that could be, in part, attributed to methodological differences in immunosuppression protocols and laboratory measurement protocols utilized in the present study. Additionally, the predictive capability of IL-2 was consistently outperformed by other tested markers, such as IL-8 [34], interferon-γ and IL-17 [36] and IL-2R in our study, thus indicating that certain limitations may apply in the utility of IL-2 as a reliable predictive marker.

On the other hand, our findings on the utility of IL-2R are consistent with the trends observed in the literature. Namely, Hagras et al. [37] observed that the mean levels of IL-2R in clinically confirmed AR(+) patients were significantly higher compared to stable, AR(-) patients (14.8 ± 6.54 ng/mL versus 6.44 ±1.95 ng/mL, p<0.001); a trend that persisted in the histologically confirmed AR cases as well (16.19 ± 7.48 ng/mL, p=0.032). Mehta et al. [38] also reported higher levels of IL-2R in recipients with severe rejection episodes compared to stable ones (1,515±496 U/mL versus 698± 333 U/mL, p=0.034). They also noted a correlation between the ratio of post- and pre-Tx IL-2R levels and the prediction of AR, with ratios of 0.6 or higher being a prognostic factor for the occurrence of AR [38]. These findings were further confirmed by García-Roca et al. [39], who noted that serum IL-2R levels were significantly higher in patients with AR compared to both patients with graft dysfunction of other aetiology and stable, AR(-) patients (6,539 ± 1,802 pg/mL versus 2,217 ± 256 pg/mL versus 2,183 ± 283 pg/mL respectively, p=0.004). All these observations are consistent with the present study, whereby, IL-2R levels were significantly higher in the AR+ group on the sixth and 14th postoperative days. In addition, there appeared to be a significant positive linear correlation between IL-2R and Cystatin-C levels, at least during the first post-Tx days. These findings are indicative of the potential of IL-2R as an alternative marker for the evaluation of renal graft function equivalent to sCr and Cystatin-C.

There are some limitations concerning this study that should be acknowledged. First of all, the small sample size of the study and the inherent imbalance of AR(+) and AR(-) groups introduce limitations to the generalizability of the observed outcomes and larger studies would need to validate these findings. In addition, the majority of patients received low-dose corticosteroids and were clinically stable throughout the duration of the study. The values of IL-2R and Cystatin-C therefore may have been partially affected by the medication. There may have also been an effect on IL-2 and IL-2R levels caused by the small differences and individualization of immunosuppressant protocol, which, given the small sample size, was not possible to assess via subanalysis. Also, the group of AR+ patients appears to have slightly elevated IL-2R levels pre-Tx compared to the AR group. Although this difference was not statistically significant, it may have affected the results of this study to some extent. It should be noted as well, that, based on the design of the study, the samples were taken at regular intervals post-Tx and not every time there was any issue with the graft. A future study, in order to surpass these limitations, would necessitate a larger sample size, with as much standardisation of immunosuppressant protocols as possible, in order to achieve case homogeneity and verify the effects observed in the present study.

## Conclusions

In conclusion, based on the results of this study, it appears that IL-2R could be considered an alternative indicator for assessing the likelihood of an AR during the early post-Tx period, offering the possibility of an early intervention to prevent the loss of the graft. However, these findings alone cannot justify the routine use of IL-2R in clinical practice and further research is necessary, with a larger sample size and more homogenous patient population in order to ascertain their validity and establish practical diagnostic and predictive thresholds.
